# Effect of Potassium-Ion-Triggered Double Helix Aggregation on Shakedown Behavior of *κ*-Carrageenan/Polyacrylamide Hydrogel

**DOI:** 10.3390/gels11060412

**Published:** 2025-05-30

**Authors:** Xueqi Zhao, Yudong Pan, Zhanrong Zhou, Yang Gao, Aijun Li, Binkai Shi, Jian Hu, Liuying Wang

**Affiliations:** 1 Rocket Force University of Engineering, Xi’an 710025, China; 18729396064@163.com (X.Z.); zhouzhou76@163.com (Z.Z.); gaoyang_nudt@126.com (Y.G.); liaijun1979@163.com (A.L.); hhusbk2009@hhu.edu.cn (B.S.); 2 The Institute of Xi’an Aerospace Solid Propulsion Technology, Xi’an 710025, China; panyudong9@foxmail.com; 3 State Key Lab for Strength and Vibration of Mechanical Structures, International Center for Applied Mechanics, Department of Engineering Mechanics, Xi’an Jiaotong University, Xi’an 710049, China

**Keywords:** fatigue damage, double helices, shakedown behavior, *κ*-carrageenan hydrogel

## Abstract

This study investigates the effect of potassium ion (K^+^) concentration on double helix aggregation in *κ*-carrageenan-based hydrogels, which significantly influences their shakedown behavior. The shakedown behavior of *κ*-carrageenan/polyacrylamide (PAAm) hydrogels was characterized by the evolution of maximum stress and energy dissipation during cyclic load. The experimental results indicate that higher K^+^ concentrations significantly improve the maximum stress in the steady state, but barely influence the energy dissipation in the steady state. The improved maximum stress can be explained by the higher density of double helix aggregation. The steady energy dissipation elucidates that the K^+^ concentration does not affect the breaking–recovering balance of the sacrificial network in cyclic loading. These results provide mechanistic insights into how ion-triggered double helix aggregation influences the shakedown behavior of *κ*-carrageenan-based hydrogels.

## 1. Introduction

In recent years, tough hydrogels have demonstrated remarkable potential in biomedical tissue engineering [1], soft robotics [2], and stretchable electronics [3], yet their broader utility hinges on long-term mechanical durability under cyclic loading [4]. Under cyclic loading, the mechanical properties of tough hydrogels degrade significantly, and the toughening mechanism gradually fails [5]. As a result, exceptional toughness cannot be equated with reliable fatigue performance [6].

Over the past years, the fatigue behavior of hydrogels has drawn much attention. Suo et al. investigated the fatigue damage behavior of PAAm/Ca-alginate tough hydrogels and defined their shakedown behavior [7,8]. These tough hydrogels exhibit shakedown behavior under cyclic loading, where the stress–strain curves of the tough hydrogel continuously evolved under cyclic loading until reaching a steady state after thousands of cycles [9]. The evolution of these curves corresponded to the progressive rupture of internal ionic bonds [10,11]. Bai et al. studied the fatigue behavior of PAAm/poly(vinyl alcohol) tough hydrogels, demonstrating that hydrogen bonding dissipates gradually after thousands of loading–unloading cycles and then recovers after 3 min [8]. In addition, covalent bonds, hydrophobic interactions, dipole–dipole interactions, and host–guest interactions are common sacrificial bonds used for toughening mechanisms [10]. Since different hydrogel systems use various toughening mechanisms, systematic experiments are necessary to study their fatigue properties [12].

Carrageenan is a hydrophilic colloid derived from red algae. As a negatively charged polysaccharide, the gelation of carrageenan is significantly influenced by the shielding effect of anions [13,14]. The type and concentration of counterions (cations) in aqueous solution can modulate the double helix formation and aggregation behavior of carrageenan, resulting in diverse three-dimensional network structures with varying junction densities and sizes [15,16,17]. For example, potassium ions (K^+^) bind to sulfate groups in *κ*-carrageenan molecules, inducing double helix aggregation [18,19].

As a naturally occurring polysaccharide, carrageenan exhibits immense potential in diverse fields, including biomedical applications [20,21], food processing [22,23], and flexible wearable electronics [24,25]. Conventional *κ*-carrageenan hydrogels, in the presence of various cations, are typically weak and brittle materials with an elastic modulus ranging from 1 to 10 Pa [26,27]. Over the past two decades, numerous energy dissipation strategies have been developed to design strong and tough *κ*-carrageenan-based hydrogels, particularly through the implementation of sacrificial network structures [12,28,29]. Furthermore, their remarkable recoverability at room temperature and their thermally induced self-healing properties have been systematically investigated [30,31]. However, the fatigue behaviors of *κ*-carrageenan-based hydrogels, where cations serve as physical crosslinkers, have not been sufficiently studied [32]. The anti-fatigue properties of *κ*-carrageenan-based hydrogels significantly impact the accuracy, reliability, and stability of their applications [33,34]. Investigating the effects of cations on the fatigue behavior of *κ*-carrageenan-based hydrogels is essential [35].

In this study, we prepared the *κ*-carrageenan/PAAm hydrogel with various K^+^ concentrations. Next, uniaxial tensile tests and cyclic loading–unloading tests were conducted. Then, the shakedown behavior of the hydrogels was characterized by the evolution of the maximum stress and energy dissipation during cyclic load. Finally, the effect of K^+^-triggered double helix aggregation on the fatigue damage behavior of *κ*-carrageenan/PAAm hydrogels was investigated.

## 2. Results and Discussion

### 2.1. Tensile Mechanical Properties

In the carrageenan family, *κ*-carrageenan is a linear polymer composed of alternating α-(1–3)-D-galactose-4-sulfate and β-(1–4)-3,6-anhydro-D-galactose units [36]. The gelation process of *κ*-carrageenan-based hydrogels in K^+^ solution, driven by the shielding effect of K^+^, begins with a transition from coils to double helices by hydrogen bonding, followed by their aggregation. Previous studies have shown that the double helix aggregation of *κ*-carrageenan is crosslinked by hydrogen bond during cooling, while being induced by the shielding effect of K^+^, and the aggregation density is dependent on the K^+^ concentration [37,38,39].

**Figure 1:**
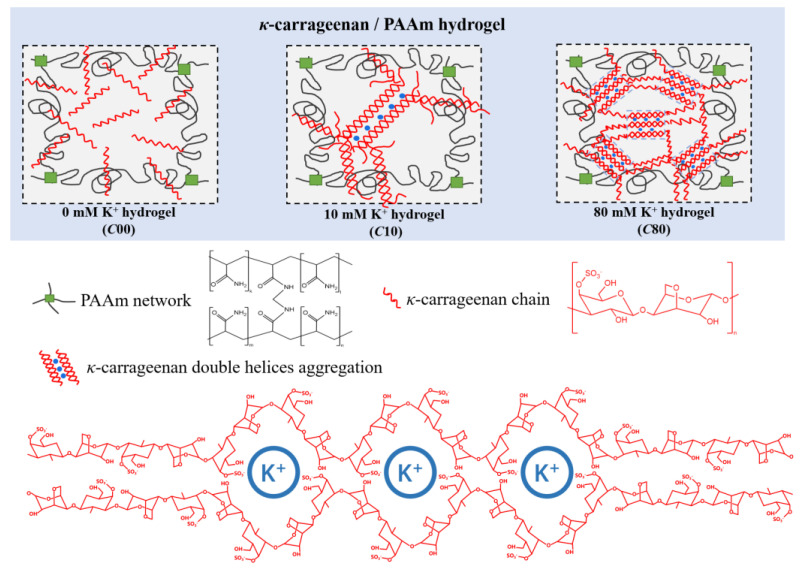
The schematics of the network structure in hydrogel without K^+^ cations (C00), with 10 mM K^+^ cations (C10), and with 80 mM K^+^ cations (C80).

In the study, we prepared a *κ*-carrageenan/PAAm hydrogel with a K^+^ concentration ranging from 0 to 80 mM. In these hydrogels, PAAm forms a chemically crosslinked network, while K^+^ cations bind to SO^3−^ groups on *κ*-carrageenan chains, triggering double helix aggregation and facilitating the crosslinking structures of double helix aggregation. Figure 1 shows the schematics of *κ*-carrageenan/PAAm hydrogels with different KCl concentrations (C00, C10, and C80), highlighting its effect on the configurational transition of *κ*-carrageenan chains and double helix aggregation behavior. The structural characteristics, such as the length of aggregated regions and the number of helices bundled within each crosslink, were determined by the K^+^ cation concentration. In fact, due to randomness in the aggregation process, the length, number, and strength of double helix aggregations, triggered by K^+^, have a wide distribution [26,28].

**Figure 2:**
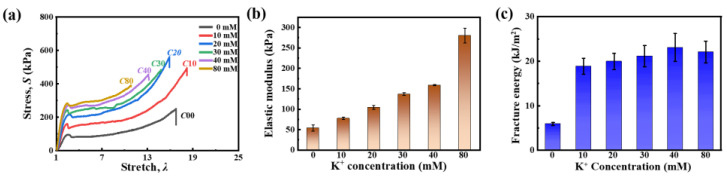
Tensile mechanical properties of the *κ*-carrageenan/PAAm hydrogel with various K^+^ concentrations. (**a**) Stress–stretch curves of hydrogel with varying K^+^ cation concentrations, labeled as C00, C10, C20, C30, C40, and C80, respectively. (**b**) Effects of K^+^ concentration on elastic modulus. (**c**) Effects of K^+^ concentration on fracture energy.

The stress–stretch curves of a *κ*-carrageenan-based hydrogel with KCl concentrations ranging from 0 to 80 mM demonstrate a significant enhancement in strength, indicating that K^+^ addition strengthens the double helix aggregation crosslinked network (Figure 2a). Especially, the modulus increased, respectively, from 53.80 ± 7.86 kPa (C00) to 77.70 ± 3.20 kPa (C10), 104.53 ± 4.52 kPa (C20), 137.07 ± 3.43 kPa (C30), 137.07 ± 3.43 kPa (C40), and 280.20 ± 17.87 kPa (C80) (Figure 2b). The fracture energy of the pure *κ*-carrageenan/PAAm hydrogel was only ~5 kJ/m^2^ (Figure 2c). In contrast, the hydrogel with added K^+^ ions consisted of *κ*-carrageenan double helix aggregates and achieved a significantly higher fracture energy of ~20 kJ/m^2^. This enhancement was attributed to the increased density of double helix aggregates. Interestingly, the fracture energy of sample C80 showed a slight decrease with increasing K^+^ concentration. This phenomenon can be explained by the fact that the higher density of double helix aggregation reduced the extensibility of the crosslinked network, consequently leading to a lower rupture stretch.

**Figure 3:**
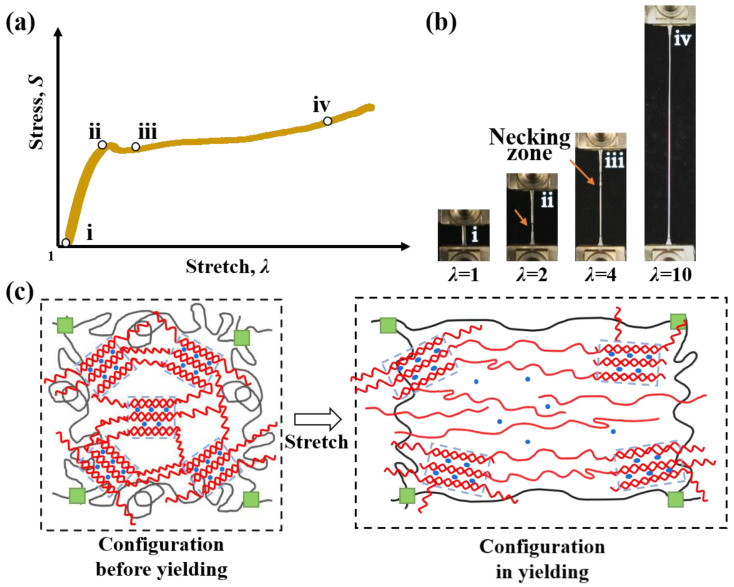
The yield behavior of the *κ*-carrageenan/PAAm hydrogel during the uniaxial tensile test. (**a**) The stress–stretch curve of C80 hydrogels. (**b**) Pictures demonstrating the yielding phenomenon during the tensile test of C80. Labels i–iv represent the correspondence between the stress–stretch curve and the picture. (**c**) Illustration of the C80 network structure before yielding and during yielding.

Moreover, a yielding phenomenon was observed during the uniaxial tensile test of these hydrogels. For example, in C80 samples, the data point labeled ii on the stress–stretch curve corresponds to the yield point, where the necking zone appears (Figure 3a). At the yield point, the primary *κ*-carrageenan double helix aggregation network begins to break into small clusters. During yielding propagation, the neck zone expands and a plateau region appears in the stress–stretch curve, indicating that *κ*-carrageenan clusters behave as sliding crosslinkers within the PAAm network. After yielding propagation, the gel softens significantly and sustains a large elongation due to the stretching of the loosely crosslinked PAAm network under stress (Figure 3b). With an increasing K^+^ cation concentration, the yield stress increased, while the yield stretch remained nearly constant at approximately 2.5. The mechanism of the yielding behavior can be interpreted as follows (Figure 3c). Below the yield point, the configuration is a double helix aggregated *κ*-carrageenan network. Subject to a load, the low-density aggregation begins to break, but the network remains intact. Above the yield point, low-density aggregation is mostly dissipated, leaving high-density aggregation clusters. The clusters are connected by long chains of *κ*-carrageenan. As the load continues to increase, the clusters slide along the long chains, exhibiting a necking phenomenon macroscopically. To investigate the shakedown behavior of the double helix aggregation network under cyclic loading, we selected the stretch below the yield point as the maximum stretch (1.5 and 2) during fatigue damage tests.

### 2.2. Viscoelastic Behavior

**Figure 4:**
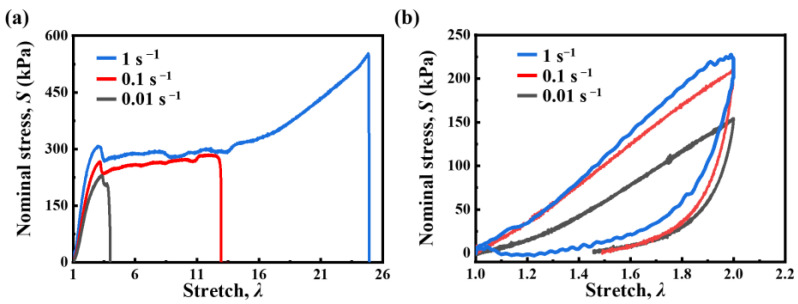
Viscoelastic behavior of the C80 hydrogels. (**a**) Stress–stretch curves measured at different loading rates. (**b**) Cyclic loading–unloading tests performed at different loading rates.

*κ*-carrageenan/PAAm hydrogels have sacrificial bonds, such as hydrogen bonds and double helix aggregation. The sacrificial bond can break and recover, endowing the potential to enhance fatigue resistance. We selected the C80 samples, which have more abundant double helix aggregation than the other samples, to investigate the viscoelastic properties of *κ*-carrageenan/PAAm hydrogels. As shown in Figure 4a, the rate-dependent behavior of hydrogels, was evaluated through tensile tests conducted at varying loading rates: 1 s^−1^, 0.1 s^−1^, and 0.01 s^−1^. The stress–stretch curves reveal that samples stretched to a higher loading rate display an enhanced modulus and improved stretchability. This is because the high loading rate limits the rearrangement of the double helix aggregation and relaxation of the polymer chains. Figure 4b illustrates the loading–unloading cycles at an applied stretch of 2.0 with varying loading rates, 1 s^−1^, 0.1 s^−1^, and 0.01 s^−1^. The hysteresis area of the stress–stretch curves increases as the loading rate increases. According to Figure 1, elastic deformation occurs in the PAAm network when the stretching ratio is 2.0. Therefore, the hysteresis originates from the breaking and recovering of double helix aggregation within the crosslinked *κ*-carrageenan network. A lower loading rate allows more time for rearrangement, resulting in reduced hysteresis. To highlight the changes in hysteresis during the shakedown process, we selected a loading rate of 1 s^−1^ for the fatigue damage test, focusing on the effect of potassium ion concentration on shakedown behavior during the fatigue of *κ*-carrageenan/PAAm hydrogel.

### 2.3. Cyclic Fatigue Damage and Shakedown Behavior

**Figure 5:**
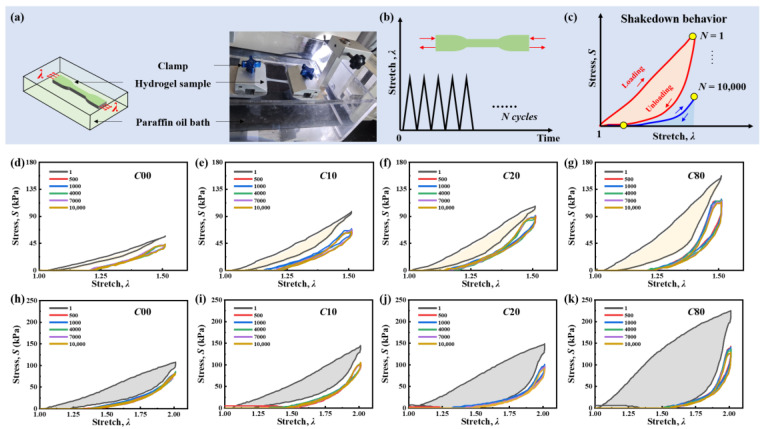
Cyclic fatigue damage of *κ*-carrageenan/PAAm hydrogel. (**a**) Experimental setup of fatigue test. (**b**) The loading curve of fatigue test, where the applied stretch λ is plotted as a function of cycles *N*. (**c**) Shakedown behavior, which involves stress *S* versus stretch λ curves over *N* cycles of the applied stretch. (**d**–**k**) Fatigue damage test of the *κ*-carrageenan/PAAm hydrogel under cyclic loads with maximum stretches of *λ*_max_ = 1.5 and *λ*_max_ = 2.0. The stress–stretch curves of (**d**,**h**) C00, (**e**,**i**) C10, (**f**,**j**) C20, and (**g**,**k**) C80.

The double helix aggregation network acts as a sacrificial network during cyclic deformation. To investigate the relationship between the density of double helix aggregation and shakedown behavior, we established an experimental setup to maintain stable water content in the hydrogels during the fatigue test (Figure 5a). It is worth mentioning that the samples used were not fully swollen. Once submersed in water bath, the hydrogel sample will imbibe water and swell, which influences its mechanical behavior greatly. To fix the water content in samples, we performed the cyclic loading–unloading test with the samples submersed in an oil bath. The hydrogel samples were then subjected to cyclic loading to obtain the stress–stretch curves (Figure 5b). The stress–stretch curves evolved cyclically and reached a stable state after hundreds of cycles, demonstrating the shakedown phenomenon (Figure 5c). We characterized the cyclic fatigue of the *κ*-carrageenan/PAAm hydrogel by applying cyclic loads to the uncut samples. The evolution of the stress–stretch curves for C00 (Figure 5d,h), C10 (Figure 5e,i), C20 (Figure 5f,j), and C80 (Figure 5g,k) are recorded as the number of cycles at a prescribed maximum stretch *λ*_max_ ranging from 1.5 to 2.0. All of the fatigue tests were subjected to 10,000 cycles in a paraffin oil bath.

**Figure 6:**
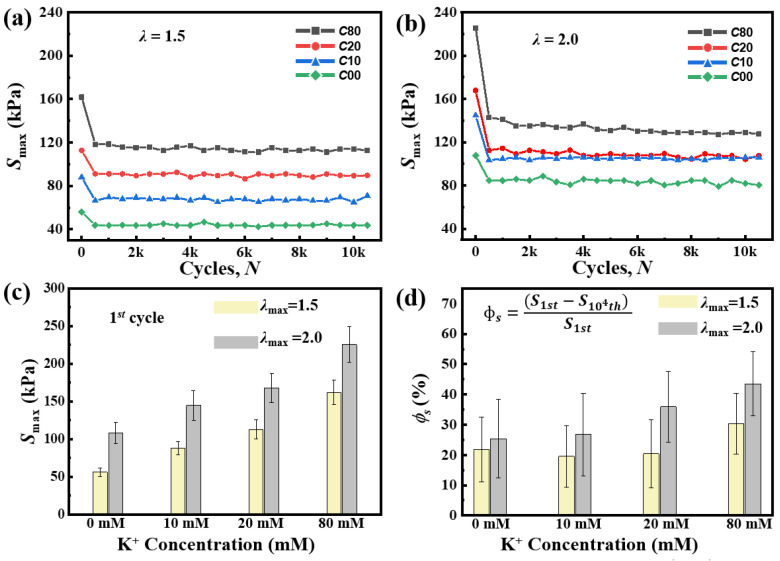
The evolution of the maximum stress *S*_max_ in the shakedown behavior of a *κ*-carrageenan/PAAm hydrogel. (**a**,**b**) *S*_max_ of the C00, C10, C20, and C80 samples in each cycle under the maximum stretches *λ*_max_ = 1.5 and *λ*_max_ = 2.0. (**c**) *S*_max_ measured during the 1st cycle of the cyclic load. (**d**) The shakedown ratio of *S*_max_, *ϕ_S_*, of these hydrogels.

**Figure 7:**
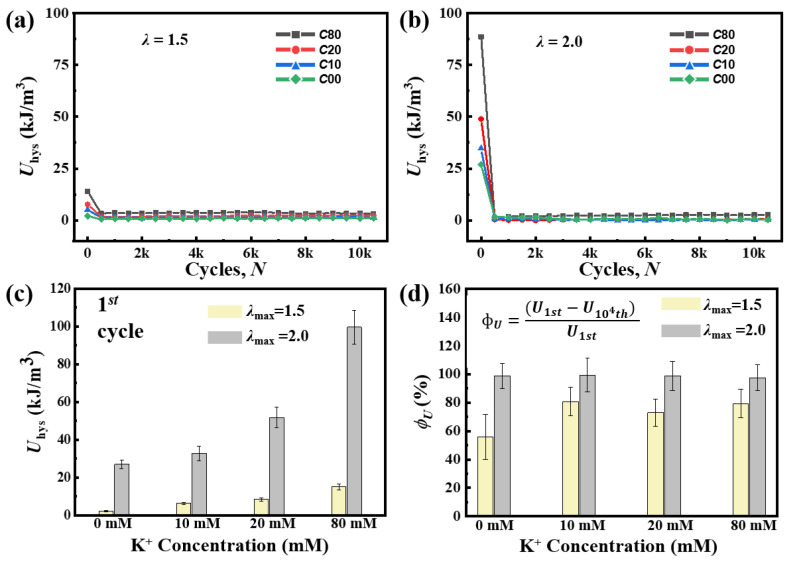
The evolution of the energy dissipation *U*_hys_ in the shakedown behavior of a *κ*-carrageenan/PAAm hydrogel. (**a**,**b**) *U*_hys_ of C00, C10, C20, and C80 samples in each cycle under the maximum stretches *λ*_max_ = 1.5 and *λ*_max_ = 2.0. (**c**) *U*_hys_ measured during the 1st cycle of the cyclic load. (**d**) The shakedown ratio of *U*_hys_, *ϕ_U_*, of these hydrogels.

To analyze the shakedown behavior, we measured the maximum stress *S*_max_ and the value of energy dissipation *U*_hys_. And then we plotted the *S*_max_-*N* curves and the *U*_hys_-*N* curves as a function of *N*. During the whole cyclic loading, all of the *S*_max_-*N* and *U*_hys_-*N* curves show shakedown behavior and reach a steady state after the first several cycles (Figure 6 and Figure 7).

The maximum stress *S*_max_ is used to characterize the density of double helix aggregation crosslinked points. In Figure 6a,b, C80 always shows a larger maximum stress *S*_max_ than the other samples, indicating that the increase in K^+^ concentration can increase the density of double helix aggregation. In the first cycle, the maximum stress *S*_max_ of C80 reached 161.77 ± 16.23 kPa at *λ*_max_ = 1.5 (Figure 6c). The *S*_max_ values increased from 56.05 ± 5.99 kPa for C00 to 88.01 ± 8.87 kPa for C10, and to 112.67 ± 12.77 kPa for C20. The increase in K^+^ concentration leads to changes in the density of double helix aggregation, resulting in the *S*_max_ increase. When *λ*_max_ is 2.0, the *S*_max_ also becomes larger because both the double helix aggregation and chemical crosslinking points are further broken. Meanwhile, the C80 attains a higher shakedown ratio of *S*_max_ (*ϕ_S_*) than the other samples and exhibits a more obvious stress softening behavior (Figure 6d). This is because of the incomplete reforming of double helix aggregation in the C80 sample within the short time scale of one loading cycle.

The value of energy dissipation *U*_hys_ is used to characterize the breakage of the aggregation structure of double helix aggregation for each cycle. The *U*_hys_ assessed by the area of the hysteresis loop exhibits an increasing trend with the elevation of K^+^ concentration during the initial cycle, as depicted in Figure 7a,b. When *λ*_max_ is 2.0, the *U*_hys_ of C00 rises sequentially from 27.04 ± 2.35 kJ/m^3^ to 32.89 ± 3.85 kJ/m^3^ for C10, then to 51.80 ± 5.33 kJ/m^3^ for C20, and finally to 99.62 ± 9.08 kJ/m^3^ for C80 during the first cycle (Figure 7c). This increasing trend is attributed to the fact that a higher concentration of K^+^ ions leads to the length of the aggregated region and the number of helices bundled within each crosslink increasing. After the first several cycles, the *U*_hys_ of all samples stay in a low state, approximately only a few kJ/m^3^, indicating that the K^+^ concentration does not affect the breaking–recovering balance of the sacrificial network in the following cycles. The shakedown ratio of *U*_hys_ (*ϕ_U_*) of all samples for *λ*_max_ = 2.0 is indeed larger than that for *λ*_max_ = 1.5 (Figure 7d), indicating that as the applied maximum stretch increases, the number of broken double helix aggregations for each cycle also increases correspondingly. When *λ*_max_ is 2.0, the value of *ϕ_U_* exceeds 95%, which does not mean that the double helix aggregation in the gel was completely consumed. The lower and steady energy dissipation elucidates that a small amount of double helix aggregation reached a breaking–recovering balance in cyclic loading.

## 3. Conclusions

In this study, fatigue damage tests of *κ*-carrageenan/PAAm hydrogels were conducted and the effect of K^+^-triggered double helix aggregation on shakedown behavior was investigated. The results of uniaxial tensile tests showed an enhanced elastic modulus, yield stress, and fracture energy with K^+^ concentration, indicating the increased density of double helix aggregation. Viscoelasticity studies highlighted the response to loading rates, with higher rates increasing the hysteresis. The shakedown behavior under cyclic loads was characterized by maximum stress and energy dissipation. This research indicated that higher K^+^ concentrations significantly improve the maximum stress in the steady state, but barely influence the energy dissipation in the steady state. This concentration–stability relationship directly informs the strategic selection of ionic environments for hydrogel-based devices subjected to cyclic loading, such as cartilage replacements or flexible sensors requiring long-term durability. These findings provide valuable insights for the development of such materials for applications that require high mechanical strength and fatigue resistance.

## 4. Materials and Methods

### 4.1. Materials

All chemicals were purchased from commercial suppliers and used directly without further purification. To form the physical network in the hydrogel, *κ*-carrageenan (Aladdin Reagent Co., Ltd., Shanghai, China, C121013) was crosslinked by potassium chloride (KCl; Aladdin Reagent Co., Ltd., Shanghai, China, P112133). To prepare the chemical network, the monomer acrylamide (AAm; Macklin Reagent Co., Ltd., Shanghai, China, A800656) was initiated by a UV-initiator 2-hydroxy-4′-(2-hydroxyethoxy)-2-methylpropiophenone (Aladdin Reagent Co., Ltd., Shanghai, China, H137984) and crosslinked by N,N’-methylenebisacrylamide (MBAA; Aladdin Reagent Co., Ltd., Shanghai, China, M128783).

### 4.2. Sample Preparation

Preparation of *κ*-carrageenan/PAAm hydrogel. The physically and chemically crosslinked *κ*-carrageenan/PAAm hydrogels were fabricated through a one-pot two-step method. Firstly, *κ*-carrageenan and monomer acrylamide were dissolved with a weight ratio of 1:9 in DI water at 90 °C, in which the total concentration of *κ*-carrageenan and monomer acrylamide was fixed at 20 wt%. Subsequently, KCl with concentrations of 0, 10, 20, 30, 40, 60, and 80 mmol/L, MBAA of 0.6 mmol%, and UV-initiator of 60 mmol% based on the monomer acrylamide were added into the aqueous solution of *κ*-carrageenan and acrylamide. The mixture was stirred magnetically at 90 °C for 2 h to obtain a homogeneous precursor solution. The solution was then poured into a pre-heated glass mold with dimensions of 130 mm × 130 mm × 2 mm. The sandwiched mold consisted of a silicone spacer with a thickness of 2 mm and two pieces of glass plates. The cast samples were then cooled at 4 °C for 30 min to form the first physically crosslinked *κ*-carrageenan network. Finally, the samples in the mold were placed under a UV lamp (wavelength of 365 nm and intensity of 160 W/cm^2^). After polymerization, the hydrogels were removed from the glass mold and then used for the following tests. C represents the concentration of K^+^ in the aqueous system. Hereafter, the gel samples prepared with K^+^ at concentrations of 0, 10, 20, 30, 40, and 80 mmol/L are referred to as C00, C10, C20, C30, C40, and C80, respectively. This paper focuses on the effect of K^+^ concentration on the shakedown behavior of hydrogels. The concentration of both the *κ*-carrageenan network and PAAm network is fixed in the material preparation.

### 4.3. Measurement

#### 4.3.1. Tensile Test

We used a tensile machine (AGS-X, Shimadzu Corporation, Taiwan, China) with a 100 N load cell. The prepared material was punctured into dumbbell-shaped samples of 35 mm in length, 2 mm in width, and 12 mm of gauge length. Both ends of the sample were held by two clamps, with the lower one being fixed. The upper clamp was pulled by the load cell at a constant rate of 100 mm/min. The stress S was calculated by dividing the applied force F by the original cross-sectional area of the specimen. The stretch ratio λ was derived by dividing the current length l during the test by the original length l_0_, λ = l/l_0_. The elastic modulus was determined by calculating the average slope of the stress–stretch curve over the stretch ratio interval of 1.0 to 1.3.

#### 4.3.2. Pure Shear Test

**Figure 8:**
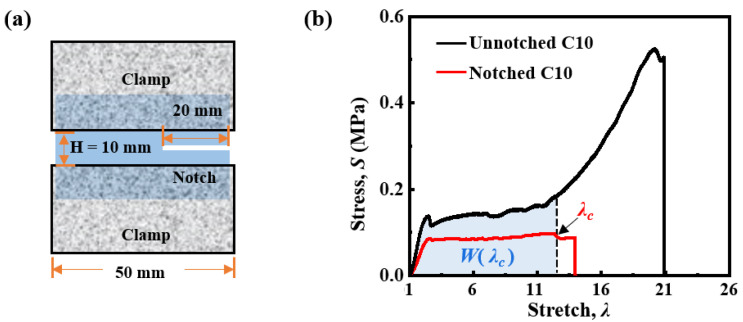
Pure shear test of *κ*-carrageenan/PAAm hydrogel. (**a**) The geometry of a sample for pure shear test. (**b**) The stress–stretch curves of pure shear samples with a notch and without a notch. The sample with a notch ruptures at a critical stretch *λ*_c_. The energy density *W*(*λ*_c_) at the critical stretch is obtained from the area under the stress–stretch curve of the sample without a notch.

The geometry of a pure shear sample with a notch is shown in Figure 8a. The thickness of each sample was 2 mm. The notch was prepared by a fresh razor blade. The pure shear samples without a notch had the same geometry in height *H*, width, and thickness as those with a notch.

A sample without a notch was firstly loaded all the way to rupture and the stress–stretch curve is obtained (Figure 8b). Then, another sample with a notch was loaded and the stress–stretch curve was recorded until the onset of crack propagation at a critical stretch *λ*_c_. The energy density *W*(*λ*_c_) was calculated as the area under the stress–stretch curve of the sample without a notch at *λ*_c_. The fracture energy of samples was calculated from

(1) $$\Gamma =W(\lambda_{c})H$$

where *H* is the height of the pure shear sample.

#### 4.3.3. Fatigue Test

The test was conducted under displacement-controlled loading conditions. Following the previous studies on the fatigue properties of hydrogel, we set the applied minimum stretch as 1.0 and varied the maximum stretch as 1.5 and 2.0. In all cyclic loading tests, the total number of cycles was set to 10,000. The loading–unloading frequency was fixed at 0.5 Hz. The stress–stretch curves were recorded every 500 cycles. To prevent hydrogel dehydration throughout the testing process, all cyclic loading experiments were conducted with samples submerged in a paraffin oil bath.

The shakedown ratio of the maximum stress *ϕ*_S_: The maximum stress, *S*_max_, was determined from the point of maximum stretch during a single loading–unloading cycle. The *ϕ*_S_ was calculated by the following equation:

(2) $$\phi_{S}=\frac{\left(S_{1st}-S_{10^{4}th}\right)}{S_{1st}}$$

where S1st is the maximum stress at the first cycle, and *S*_10_4th is the maximum stress at the 10,000th cycle.

The shakedown ratio of the energy dissipation *ϕ_U_*: The energy dissipation, *U*_hys_, was determined by the area of each cycle. The *ϕ_U_* value was calculated by the following equation:

(3) $$\phi_{U}=\frac{\left(U_{1st}-U_{10^{4}th}\right)}{U_{1st}}$$

where *U*1st is the energy dissipation at the first cycle, and *U*_10_4th is the energy dissipation at the 10,000th value.

## Data Availability

No new data were created or analyzed in this study. Data sharing is not applicable to this article.

## References

[1] Chen X., Liu F., Yu Q., Yang M., Suo Z., Tang J. (2025). A Soft and Fatigue-Resistant Material that Mimics Heart Valves. Matter.

[2] Zhao X. (2023). A Bioadhesive Robot to Activate Muscles. Nat. Mater..

[3] Li Y., Tan S., Zhang X., Li Z., Cai J., Liu Y. (2025). Design Strategies and Emerging Applications of Conductive Hydrogels in Wearable Sensing. Gels.

[4] Protsak I.S., Morozov Y.M. (2025). Fundamentals and Advances in Stimuli-Responsive Hydrogels and Their Applications: A Review. Gels.

[5] Zhang W., Hu J., Tang J., Wang Z., Wang J., Lu T., Suo Z. (2019). Fracture Toughness and Fatigue Threshold of Tough Hydrogels. ACS Macro. Lett..

[6] Han Z., Lu Y., Qu S. (2024). Design of Fatigue-Resistant Hydrogels. Adv. Funct. Mater..

[7] Bai R., Yang Q., Tang J., Morelle X.P., Vlassak J., Suo Z. (2017). Fatigue Fracture of Tough Hydrogels. Extrem. Mech. Lett..

[8] Bai R., Yang J., Morelle X., Yang C., Suo Z. (2018). Fatigue Fracture of Self-Recovery Hydrogels. ACS Macro. Lett..

[9] Sun J., Zhao X., Illeperuma W.R.K., Chaudhuri O., Oh K., Mooney D.J., Vlassak J.J., Suo Z. (2012). Highly Stretchable and Tough Hydrogels. Nature.

[10] Bai R., Yang J., Suo Z. (2019). Fatigue of Hydrogels. Eur. J. Mech. A-Solids.

[11] Zhao X., Wu J., Zhou Y., Pan Y., Lu T., Song X., Hu J. (2021). Fatigue Behaviors of Physical Hydrogels Based on Hydrogen Bonds. Extrem. Mech. Lett..

[12] Li X., Gong J. (2024). Design Principles for Strong and Tough Hydrogels. Nat. Rev. Mater..

[13] Wang Z., Long J., Zhang C., Hua Y., Li X. (2025). Effect of Polysaccharide on Structures and Gel Properties of Microgel Particle Reconstructed Soybean Protein Isolate/Polysaccharide Complex Emulsion Gels as Solid Fat Mimetic. Carbohydr. Polym..

[14] Zabik M.E., Aldrich P.J. (1967). The Effect of Cations on the Viscosity of Lambda-Carrageenan. J. Food Sci..

[15] Liu S., Zhang H., Yu W. (2020). Simultaneously Improved Strength and Toughness in κ-Carrageenan/Polyacrylamide Double Network Hydrogel via Synergistic Interaction. Carbohydr. Polym..

[16] Miao Y., Xu M.D., Zhang L.D. (2021). Electrochemistry-Induced Improvements of Mechanical Strength, Self-Healing, and Interfacial Adhesion of Hydrogels. Adv. Mater..

[17] Núñez-Santiago M., Pérez-López A., Espinosa-Solares T., Nicolás-Vázquez M., Laureano-López B. (2023). Sol-gel transition diagram and theoretical study of κ-carrageenan in the presence of calcium ions. LWT-Food Sci. Technol..

[18] Deng Y., Huang M., Sun D., Hou Y., Li Y.B., Dong T.S., Wang X.H., Zhang L., Yang W.Z. (2018). Dual Physically Cross-Linked κ-Carrageenan-Based Double Network Hydrogels with Superior Self-Healing Performance for Biomedical Application. ACS Appl. Mater. Interfaces.

[19] Gonçalves G., Faria B., Moraes I., Hilliou L. (2025). Role of the Molecular Mass on the Elastic Properties of Hybrid Carrageenan Hydrogels. Gels.

[20] Das I.J., Bal T. (2024). Exploring Carrageenan: From Seaweed to Biomedicine—A Comprehensive Review. Int. J. Biol. Macromol..

[21] Batet D., Navarro-Segarra M., Gonçalves R., Costa C.M., Lanceros-Mendez S., Pablo Esquivel J. (2024). The Versatility of Iota-Carrageenan Biopolymer for The Fabrication of Non-Toxic bio-Based Energy Storage Devices. Chem. Eng. J..

[22] Fan Z., Cheng P., Zhang P., Gao Y., Zhao Y., Liu M., Gu J., Wang Z., Han J. (2022). A Novel Multifunctional Salecan/κ-Carrageenan Composite Hydrogel with Anti-Freezing Properties: Advanced Rheology, Thermal Analysis and Model Fitting. Int. J. Biol. Macromol..

[23] Sabu Mathew S., Jaiswal A.K., Jaiswal S. (2024). Carrageenan-Based Sustainable Biomaterials for Intelligent Food Packaging: A Review. Carbohydr. Polym..

[24] Zhang J., Tang J., Zhang J., Ren T., Wei J., Liang Y., Feng E., Han X., Ma X. (2024). An Antifreeze, Self-Healing Dual-Network Organogel for Supercapacitor and Anticounterfeiting Equipment. ACS Appl. Energy Mater..

[25] Cao L., Li X., Hu X. (2024). An Antibacterial, Highly Sensitive Strain Sensor Based on an Anionic Copolymer Interpenetrating with κ-Carrageenan. ACS Biomater. Sci. Eng..

[26] Mangione M.R., Giacomazza D., Bulone D., Martorana V., Cavallaro G., San Biagio P.L. (2005). K+ and Na+ Effects on The Gelation Properties of κ-Carrageenan. Biophys. Chem..

[27] Choudhury A.R. (2023). Self-Assembled pH-Stable Gellan/κ-Carrageenan Bigel: Rheological Studies and Viscosity Prediction by Neural Network. Int. J. Biol. Macromol..

[28] Liu S., Li L. (2016). Recoverable and Self-Healing Double Network Hydrogel Based on κ-Carrageenan. ACS Appl. Mater. Interfaces.

[29] Zhang H., Shi L., Zhou J. (2023). Recent Developments of Polysaccharide-Based Double-Network Hydrogels. J. Polym. Sci..

[30] Fan Z., Duan L., Gao G. (2020). Self-Healing Carrageenan-Driven Polyacrylamide Hydrogels for Strain Sensing. Sci. China-Technol. Sci..

[31] Mirzaei A., Javanshir S., Servati P. (2023). Thermal Insulation Properties of Lightweight, Self-Healing, and Mesoporous Carrageenan/PMMA Cryogels. RSC Adv..

[32] Safarpour F., Kharaziha M., Mokhtari H., Emadi R., Bakhsheshi-Rad H.R., Ramakrishna S. (2023). Kappa-Carrageenan Based Hybrid Hydrogel for Soft Tissue Engineering Applications. Biomed. Mater..

[33] Horinaka J., Takagaki H., Tanaka T., Takigawa T. (2022). Effects of Gelation Concentration on Cyclic Deformation Behavior of κ-Carrageenan Hydrogels. Int. J. Biol. Macromol..

[34] Alipour S., Pourjavadi A., Hosseini S. (2023). Magnetite Embedded κ-Carrageenan-Based Double Network Nanocomposite Hydrogel with Two-Way Shape Memory Properties for Flexible Electronics and Magnetic Actuators. Carbohydr. Polym..

[35] Zeng L., Liu B., Duan L., Gao G. (2023). Tough, Recyclable and Biocompatible Carrageenan-Modified Polyvinyl Alcohol Ionic Hydrogel with Physical Cross-Linked for Multimodal Sensing. Int. J. Biol. Macromol..

[36] Barbeyron T., Michel G., Potin P., Henrissat B., Kloareg B. (2000). ι-Carrageenases Constitute a Novel Family of Glycoside Hydrolases, Unrelated to That of κ-Carrageenases. J. Biol. Chem..

[37] Sun X.Y., Ye L.N., Liang H.Y. (2021). Extremely Stretchable and Tough Hybrid Hydrogels Based on Gelatin, κ-Carrageenan and Polyacrylamide. Soft Matter.

[38] Li D., Yang D., Yang X., Wang Y., Guo Z., Xia Y., Sun S., Guo S. (2016). Double-Helix Structure in Carrageenan-Metal Hydrogels: A General Approach to Porous Metal Sulfides/Carbon Aerogels with Excellent Sodium-Ion Storage. Angew. Chem. Int. Ed. Engl..

[39] Kulkarni R.V., Boppana R., Krishna Mohan G., Mutalik S., Kalyane N.V. (2012). pH-Responsive Interpenetrating Network Hydrogel Beads of Poly(acrylamide)-g-Carrageenan and Sodium Alginate for Intestinal Targeted Drug Delivery: Synthesis, in Vitro and In Vivo Evaluation. J. Colloid Interface Sci..

